# Fronto-thalamic structural and effective connectivity and delusions in schizophrenia: a combined DTI/DCM study

**DOI:** 10.1017/S0033291720000859

**Published:** 2021-09

**Authors:** Gábor Csukly, Ádám Szabó, Patrícia Polgár, Kinga Farkas, Gyula Gyebnár, Lajos R. Kozák, Gábor Stefanics

**Affiliations:** 1 Department of Psychiatry and Psychotherapy, Semmelweis University, Budapest, Hungary; 2 Magnetic Resonance Research Centre, Semmelweis University, Budapest, Hungary; 3 Translational Neuromodeling Unit (TNU), Institute for Biomedical Engineering, University of Zurich & ETH Zurich, Wilfriedstrasse 6, 8032, Zurich, Switzerland

**Keywords:** Anterior cingulum, connectivity, DCM, delusion, DTI, PEB, PANSS, schizophrenia, thalamus

## Abstract

**Background:**

Schizophrenia (SZ) is a complex disorder characterized by a range of behavioral and cognitive symptoms as well as structural and functional alterations in multiple cortical and subcortical structures. SZ is associated with reduced functional network connectivity involving core regions such as the anterior cingulate cortex (ACC) and the thalamus. However, little is known whether effective coupling, the directed influence of one structure over the other, is altered during rest in the ACC–thalamus network.

**Methods:**

We collected resting-state fMRI and diffusion-weighted MRI data from 18 patients and 20 healthy controls. We analyzed fronto-thalamic effective connectivity using dynamic causal modeling for cross-spectral densities in a network consisting of the ACC and the left and right medio-dorsal thalamic regions. We studied structural connectivity using fractional anisotropy (FA).

**Results:**

We found decreased coupling strength from the right thalamus to the ACC and from the right thalamus to the left thalamus, as well as increased inhibitory intrinsic connectivity in the right thalamus in patients relative to controls. ACC-to-left thalamus coupling strength correlated with the Positive and Negative Syndrome Scale (PANSS) total positive syndrome score and with delusion score. Whole-brain structural analysis revealed several tracts with reduced FA in patients, with a maximum decrease in white matter tracts containing fronto-thalamic and cingulo-thalamic fibers.

**Conclusions:**

We found altered effective and structural connectivity within the ACC–thalamus network in SZ. Our results indicate that ACC–thalamus network activity at rest is characterized by reduced thalamus-to-ACC coupling. We suggest that positive symptoms may arise as a consequence of compensatory measures to imbalanced fronto-thalamic coupling.

## Background

Schizophrenia (SZ) is a complex disorder characterized by a range of behavioral, cognitive, and emotional symptoms. Structural and functional alterations in multiple cortical and subcortical structures have been implicated in the neurobiology of SZ (Birur, Kraguljac, Shelton, & Lahti, 2017). A recent meta-analysis of regional brain volumes in patients with SZ suggested the anterior cingulate cortex (ACC) as a core structure affected by the disorder due to its consistently lower mean volume (Brugger & Howes, 2017). Grey matter reductions have been found to be accompanied by reductions in neuronal, synaptic, and dendritic density (Fornito, Yucel, Patti, Wood, & Pantelis, 2009). ACC volume (Choi et al., 2005; Palaniyappan, Mallikarjun, Joseph, White, & Liddle, 2011), regional cerebral blood flow (Sabri et al., 1997), dopamine D2 receptor binding (Suhara et al., 2002), and activation during reward anticipation (Walter, Kammerer, Frasch, Spitzer, & Abler, 2009) correlate with positive symptoms. Beside metabolic (Fujimoto et al., 2007) and functional (Adams & David, 2007; Nelson, Bjorkquist, Olsen, & Herbener, 2015) abnormalities, within- (Wang, Rau, Li, Chen, & Yu, 2015) and between-region connectivity of the ACC (Allen et al., 2010; Cui et al., 2015; Fletcher, McKenna, Friston, Frith, & Dolan, 1999; White, Joseph, Francis, & Liddle, 2010; Yan et al., 2012) has been found to be altered in SZ. ACC hypoactivity in patients has been associated with persecutory delusions (Blackwood et al., 2004), whereas hyperactivity in a network of prefrontal regions including the ACC has been suggested to underlie the delusions of reference (Lariviere et al., 2017). Furthermore, patients with auditory verbal hallucinations are characterized with reduced functional connectivity in neural circuitry involving the ACC (Alderson-Day, McCarthy-Jones, & Fernyhough, 2015; Chang et al., 2017; Vercammen, Knegtering, den Boer, Liemburg, & Aleman, 2010). Thus, a remarkable body of evidence indicates that ACC dysfunction in SZ might underlie or contribute to psychotic symptoms.

The fronto-thalamic circuitry has long been implicated in SZ (Andreasen, Paradiso, & O'Leary, 1998). Structural (Adriano, Spoletini, Caltagirone, & Spalletta, 2010; Byne, Hazlett, Buchsbaum, & Kemether, 2009; Dorph-Petersen & Lewis, 2017; Haijma et al., 2013; Konick & Friedman, 2001), metabolic (Soyka, Koch, Moller, Ruther, & Tatsch, 2005), and cognitive functional (Andrews, Wang, Csernansky, Gado, & Barch, 2006; Minzenberg, Laird, Thelen, Carter, & Glahn, 2009) neuroimaging studies reported alterations of the thalamus in SZ. Thalamo-cingulate connectivity, in particular, was reported using functional (Anticevic et al., 2014) and effective connectivity (Wagner et al., 2013, 2015) measures during a task, and a recent review on the role of the thalamus in SZ concluded that a growing body of evidence clearly suggests the relevance of thalamo-prefrontal interactions (Pergola, Selvaggi, Trizio, Bertolino, & Blasi, 2015).

Evidence for reduced functional ACC–thalamic connectivity in SZ was found also in the resting state (Wang et al., 2015). Resting-state functional connectivity in functional magnetic resonance imaging (rs-fMRI) is widely used to assess the intrinsic neural activity that supports cognitive functioning (e.g. Buckner, Krienen, Castellanos, Diaz, & Yeo, 2011; Yeo et al., 2011). Rs-fMRI focuses on spontaneous, low-frequency fluctuations (<0.1 Hz) in the blood oxygenation level-dependent (BOLD) signal that occur in the absence of a task or stimulus to identify functional networks by mapping coupling based on correlations between regions (e.g. Cole, Smith, & Beckmann, 2010; Lee, Smyser, & Shimony, 2013; Tomasi & Volkow, 2011). This method is particularly suited for patient studies as it may offer a better signal to noise ratio than conventional task-based approaches and allows for a broader sampling of patient populations (Fox & Greicius, 2010). Using this method, multiple networks have been found to be altered in SZ (Damoiseaux et al., 2006; Moussa, Steen, Laurienti, & Hayasaka, 2012; Sheffield & Barch, 2016), and alteration of resting-state thalamo-cortical functional connectivity has been suggested as a potential marker to distinguish patients from healthy controls (Cheng et al., 2015). Furthermore, a recent review of thalamo-cortical rsfMRI studies in SZ (Giraldo-Chica & Woodward, 2017) concluded that alterations are characterized by reduced prefrontal–thalamic connectivity.

While functional connectivity measures can demonstrate temporal correlations between brain regions, they do not allow inferring the directed influence of one region over the other. Since fronto-thalamic connections are reciprocal, to better understand the underlying cause of reduced prefrontal–thalamic connectivity in SZ, the causal impact one region has over the other should be considered. Dynamic causal modeling (DCM) (Friston, 2009; Friston et al., 2017) has been widely used for modeling casual interactions in neuroimaging data.

Diffusion tensor imaging (DTI) is often used to study structural neural connectivity. It is unique in its ability to assess *in vivo* the structural integrity of white matter (WM) fiber bundles (Pierpaoli, Jezzard, Basser, Barnett, & DiChiro, 1996). DTI studies of SZ revealed abnormalities in WM tracts in several areas, which are already present at the early stages of the disease (for a review, see Samartzis, Dima, Fusar-Poli, & Kyriakopoulos, 2014). Combining fMRI and DTI allows assessing both functional and structural connectivity in the same patient. Studies employing both techniques mostly suggest altered connectivity involving frontal regions in SZ (Ellison-Wright & Bullmore, 2009; Fitzsimmons, Kubicki, & Shenton, 2013; Wagner et al., 2013, 2015).

Several lines of evidence suggest that SZ is associated with connectivity reductions involving frontal structures (Pettersson-Yeo, Allen, Benetti, McGuire, & Mechelli, 2011). While previous studies demonstrated reduced prefrontal–thalamic functional coupling, here we focus on the effective connectivity between a core prefrontal structure, the ACC, and the bilateral thalamus in resting state and investigate a potential relationship between symptom severity and coupling strength. To this end, first we estimated coupling strength between nodes of a network comprising the ACC and the left and right thalamus using DCM. Based on the study by Tomasi and Volkow (2011) on resting-state networks in a large sample of healthy individuals, we focused on the medial dorsal (MD) thalamic nuclei, since they have a central role in the so-called ‘thalamus-hub network’. Next, we explored the relationship between altered effective connectivity parameters in the SZ group and psychotic symptoms. Treatments of the dysconnection hypothesis in the predictive coding framework explain core symptoms (e.g. hallucinations and delusions) as false inference involving disrupted message passing between various levels of cortical hierarchies (Adams, Stephan, Brown, Frith, & Friston, 2013; Corlett, Taylor, Wang, Fletcher, & Krystal, 2010; Fletcher & Frith, 2009). Thus, we expected coupling strength within the ACC–thalamus network to show a relationship with symptoms. Based on previous findings, we expected decreased connectivity between the ACC and thalamic areas in patients. Furthermore, we used DTI to explore structural whole-brain connectivity to potentially link the alterations of effective connectivity to the structural integrity of WM tracts.

## Methods

### Subjects and procedures

Eighteen patients with SZ and 20 neurotypical control volunteers, with no known history of psychiatric disorder, participated in the study. The two groups did not differ in age and education (Table 1). Selection criteria for all participants were no history of any central nervous system disease, mental retardation, epileptic seizure, substance dependence or substance abuse (in 3 months prior to enrollment), and no history of head injury with loss of consciousness for more than 10 min.

**Table 1.** Demographic and clinical characteristics

	SCH *N* = 18	Control *N* = 20	χ^2^/*t* test	*p* value
Age, years, mean (s.d.^a^)	35.4 (7.18)	35.5 (8.82)	*t* = 0.04	0.97
Gender, *n* (%)				
Female	8 (44.4)	7 (35.0)	χ^2^ = 0.3	0.73
Male	10 (55.6)	13 (65.0)		
Education, years, mean (s.d.)	15.0 (3.37)	16.8 (2.96)	*t* = 1.7	0.09
Employed/student	62.5%	100%	χ^2^ = 7.4	*p* = 0.007*
Lives in his/her own flat/house	82.4%	100%	Fisher's exact test	0.23
Married	12.5%	37.5%	χ^2^ = 2.7	*p* = 0.11
Satisfaction with life (0 = entirely unsatisfied; 4 = neutral; 7 = entirely satisfied), mean (s.d.)	4.8 (1.2)	5.3 (0.6)	*t* = 1.6	*p* = 0.12
Pharmacotherapy dose, mg/day, mean (s.d.)				
Benzodiazepines^b^	0.6 (1)	–		
Antipsychotics^c^	648.64 (282.1)	–		
PANSS total^d^, mean (s.d.)	76.1 (16.9)	–		
PANSS positive, mean (s.d.)	16.4 (5.8)	–		
PANSS negative, mean (s.d.)	20.5 (6.9)	–		
PANSS general, mean (s.d.)	39.1 (8.6)	–		
Illness duration, years, mean (s.d.)	11.4 (6.9)	–		

a Standard deviation.

b Clonazepam equivalent.

c Chlorpromazine equivalent.

d PANSS, Positive And Negative Syndrome Scale.

* Significant between-group difference.

All patients met the criteria for SZ based on the Structured Clinical Interview for Diagnostic and Statistical Manual of Mental Disorders, 4th Edition (DSM-IV) (American Psychiatric Association, 1994). In the SZ group, the Positive and Negative Syndrome Scale (PANSS) was administered by a trained psychiatrist (Kay, Fiszbein, & Opler, 1987). At the time of testing, all patients were taking antipsychotic medication with a mean chlorpromazine equivalent dose of 648.6 mg/day (Gardner, Murphy, O'Donnell, Centorrino, & Baldessarini, 2010) and benzodiazepines with a 0.6 mg/day clonazepam equivalent. All participants gave written informed consent. The study was carried out at the Semmelweis University, Department of Psychiatry and Psychotherapy, Budapest, Hungary and was approved by the Institutional Ethics Board. Demographics and the characteristics of participants are shown in Table 1.

### Image acquisition

Image acquisitions were done at the MR Research Center, Semmelweis University on a 3 Tesla Philips Achieva whole-body MRI scanner (Philips Medical Systems, Best, The Netherlands) equipped with an eight-channel SENSE head coil.

The high-resolution, whole-brain anatomical images were obtained using a T1-weighted three-dimensional spoiled gradient echo (T1W 3D Turbo Field Echo) sequence. A total of 180 contiguous slices were acquired from each subject with the following imaging parameters: TR (time resolution) = 9.7 ms; TE (echo time) = 4.6 ms; flip angle = 8°; FOV (field-of-view) of 240 mm × 240 mm; voxel size of 1.0 × 1.0 × 1.0 mm.

Brain diffusion-weighted MRI images were collected with a single-shot spin-echo echo-planar imaging (EPI) sequence, with *b* = 800 s/mm^2^ diffusion weighting in 32 directions and one *b* = 0 image. In-plane resolution was 1.67 × 1.67 mm; whole-brain coverage was achieved with 70, 2 mm-thick axial slices with no gap; TR = 9660 ms repetition time, TE = 75.6 ms echo time, and 90° flip angle was used. The total acquisition time was 8:32 min.

The ‘resting state’ part of the fMRI acquisition took approximately 8.5 min. During that time, subjects were instructed to fixate on a cross in the center of the screen. Subjects were briefed whether they fell asleep during the recording process, and no subject reported doing so. Head motion was minimized using foam padding. Functional images were acquired using a T2* weighted EPI sequence with the following parameters: TR = 2.0 s; TE = 30 ms; flip angle = 70°; FOV of 240 mm × 240 mm; voxel size of 3.0 × 3.0 × 4.0 mm; number of slices = 36.

### Preprocessing for rsfMRI

Image preprocessing was performed using Statistical Parametric Mapping (SPM12, v7219; RRID: SCR_007 037; Wellcome Department of Cognitive Neurology, London, UK) in Matlab (Mathworks, Natick, MA, USA). Preprocessing of functional images of each subject included slice timing correction, realignment, and normalization into a standard template (Montreal Neurological Institute, MNI). Normalized images were smoothed in space with an 8 mm full-width at half-maximum 3D isotropic Gaussian kernel and high-pass filtered (128 s, ~0.008 Hz) to remove low-frequency drifts. Higher frequencies (>0.1 Hz) were not removed, since they are known to contain meaningful information in resting-state studies (Feinberg et al., 2010; Lin et al., 2015). Furthermore, fMRI time series were preprocessed by regressing out the effects of six rigid motions and the principal components of signal fluctuations in WM and cerebrospinal fluid masks.

### DCM for cross-spectral density

Our analysis consisted of the following steps at the first (subject) level: region of interest (RoI) specification, extraction of time series, model specification, and estimation of model parameters. We defined RoIs as the spheres of 7 mm radius centered on voxels in the ACC, and the left and right thalamus that showed strong functional connectivity in a previous study that investigated resting-state networks on a sample of >900 healthy subjects (Tomasi & Volkow, 2011). Talairach coordinates: ACC [–1 12 38], the left [–12 –19 8], and the right thalamus [12 –19 8]. It is worth noting that due to the location of the ACC, this ROI likely included voxels from both hemispheres.

DCM employs a neurobiologically informed model for the observed BOLD response and allows the estimation of effective connectivity, i.e. the strength and direction of interaction between nodes of the network thereby revealing causal relationships (Daunizeau, David, & Stephan, 2011). Recent DCM studies in SZ revealed abnormal effective coupling involving prefrontal regions (Deserno, Sterzer, Wustenberg, Heinz, & Schlagenhauf, 2012; Wagner et al., 2013, 2015; Zhou et al., 2018). In DCM, for cross-spectral densities (CSD) (Moran et al., 2009), neuronal activity is summarized in terms of its spectral density (when modeling a single source) or CSD (when modeling multiple sources). Spectral DCM for fMRI models coupled neuronal fluctuations within a network and identifies the effective coupling parameters that best explain functional connectivity observed in hemodynamic responses (Friston, Kahan, Biswal, & Razi, 2014; Razi, Kahan, Rees, & Friston, 2015). In the current study, we employed spectral DCM using a bilinear model with one state per region and with no stochastic effects, as it is more computationally efficient than stochastic DCM and has shown higher sensitivity to group differences for the estimation of effective connectivity parameters (Friston et al., 2014; Zeidman et al., 2019a).

**Fig. 1.**
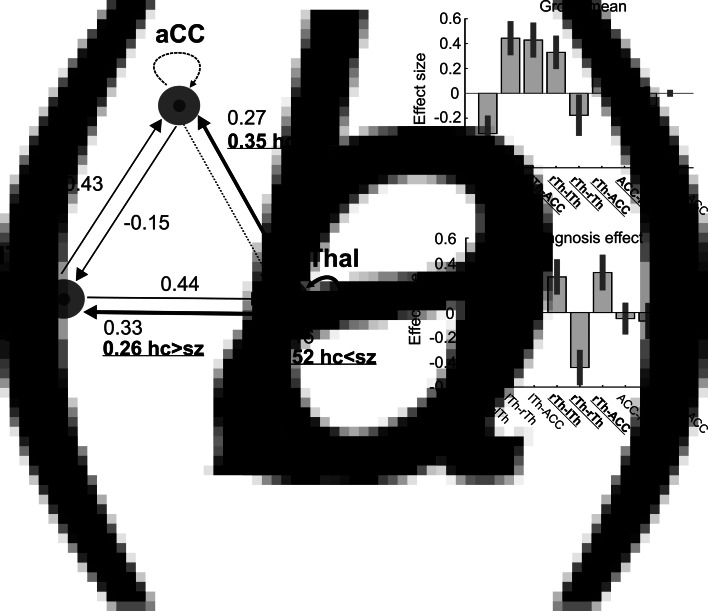
The dynamic causal model (DCM) of the thalamus hub network. (*a*) The thalamus hub network, where all lines (connections) constitute the full model we used. Solid lines show the winning model (connections with a probability >0.95), while dashed lines indicate connections not remaining in the model after PEB. Thick lines and labels in bold/underline font indicate connections differing between study groups (probability >0.95). (*b*) Estimated (posterior) connectivity parameters (effect sizes) for the whole sample (top) and for the diagnosis effect (bottom). Labels in bold/underline font show significant coupling parameters after PEB. Label abbreviations: lTh, left thalamus; rTh, right thalamus; ACC, anterior cingulate cortex.

At the second (group) level, the following procedures were applied using Parametric Empirical Bayes (PEB): Bayesian model reduction, searching over nested models, and comparison of effective connectivity parameters (Zeidman et al., 2019b). ‘Empirical Bayes refers to the Bayesian inversion or fitting of hierarchical models. In hierarchical models, constraints on the posterior density over model parameters at any given level are provided by the level above. These constraints are called empirical priors because they are informed by empirical data’ (Friston et al., 2016). The aim of the group-level analysis was twofold: (1) determine the mean connection strength across all subjects (group mean for the whole sample); (2) determine the difference in connection strengths between study groups. To this end, first we specified a full model containing all possible connections between the above described three nodes (Fig. 1). In the subsequent PEB analysis, a Bayesian model reduction was applied (Friston & Penny, 2011; Friston, Li, Daunizeau, & Stephan, 2011) which involved Bayesian inversion and comparison of models that are reduced forms of the full (or parent) model. It can be applied whenever models can be specified in terms of (reduced) prior densities. Connections between the three nodes were switched on and off in an iterative process to test their effect on free energy, an approximation of the log model evidence (Friston et al., 2016). Parameters that did not contribute to the free energy were switched off by setting their prior mean and variance to zero. Finally, a leave-one-out (LOO) method was applied to validate the group-level results (Friston et al., 2016), where a PEB model was fitted to all but one subject, and covariates for the left-out subject were predicted. This was repeated with each subject left out and the accuracy of the prediction was recorded.

### Preprocessing for DTI and diffusion tensor fitting

The ExploreDTI toolbox (Leemans, Jeurissen, Sijbers, & Jones, 2009) was used to preprocess DTI data. In order to correct for subject motion, rigid body transformation was applied, while non-rigid transformations were used to correct for susceptibility-related and EPI-induced distortions with the local rotation of the *b*-matrix (the diffusion weighting directions) to avoid angular inaccuracies (Leemans & Jones, 2009). Coordinate system transformation was also applied. The high-resolution T1-weighted images were used as templates for registration to correct the distortions inherent to the EPI-acquisition method (Jezzard, Barnett, & Pierpaoli, 1998); thereby DTI-images were spatially aligned to the T1-weighted images.

After tensor fitting, fractional anisotropy (FA), a voxel-wise DTI-measure (Alexander et al., 2011; Basser & Pierpaoli, 1996; Pierpaoli & Basser, 1996), was calculated from the tensor eigenvalues using the Robust Estimation of Tensors by Outlier Rejection (RESTORE) algorithm (Chang, Jones, & Pierpaoli, 2005). (See online Supplementary Material for details on tensor fitting and DTI scalar calculations.) Differences in FA between-study groups were analyzed by a general linear model with age and gender as covariates.

### Correlation analyses

The correlations between effective connectivity (derived from DCM of rsfMRI), DTI measures, and clinical parameters (PANSS positive and negative scores) were analyzed. Correlation between DCM coupling parameter estimates and PANSS scores were limited to connections of the winning model following Bayesian model comparison and reduction by PEB (Friston et al., 2016). If a correlation was found between the PANSS total positive or negative score and a functional connectivity measure, then the correlations between the connectivity strength and the items of the given PANSS sub-score were also analyzed. The Bonferroni correction for multiple testing was applied in case of PANSS sub-scores resulting in a corrected *α*-value of (0.05/7 = ) 0.007. Regarding DTI, only those ROIs were included in the correlation analyses where significant between-group differences were found in effective connectivity.

## Results

### Spectral DCM

We found decreased coupling strength in patients relative to controls from the right thalamus to the ACC (probability >0.99), and from the right thalamus to the left thalamus (probability >0.99), while an increased self-(inhibitory) connectivity in the right thalamus (probability >0.99) was found in patients relative to controls (Fig. 1*a*). Between-group differences were corrected for age and gender. Connectivity differences between study groups and group mean connections that survived a non-zero criterion with a posterior confidence of 95% (i.e. an effect size of zero was outside the confidence interval) are considered significant. Since only one multivariate test was used based on Bayesian statistics, correction for multiple comparisons was not necessary (Park et al., 2017). In the whole sample, all connections survived the above criteria except the ACC to rThal and the intrinsic ACC connections (Fig. 1*a*, dotted lines). Mean explained variance by the reduced model at the subject-level was 89.1% (s.d. = 11.1%) in the control group and 86.5% (s.d. = 20.6%) in the patient group. Black and red solid lines in Fig. 1*a* show connections that survived the above criterion in the whole sample (group mean). Note that intrinsic connections (curved arrows) correspond to log scaling parameters such that the positive values indicate increased inhibition whereas negative coupling values indicate decreased intrinsic inhibition relative to the prior.

### Predicting diagnostic label based on effective connectivity

**Fig. 2.**
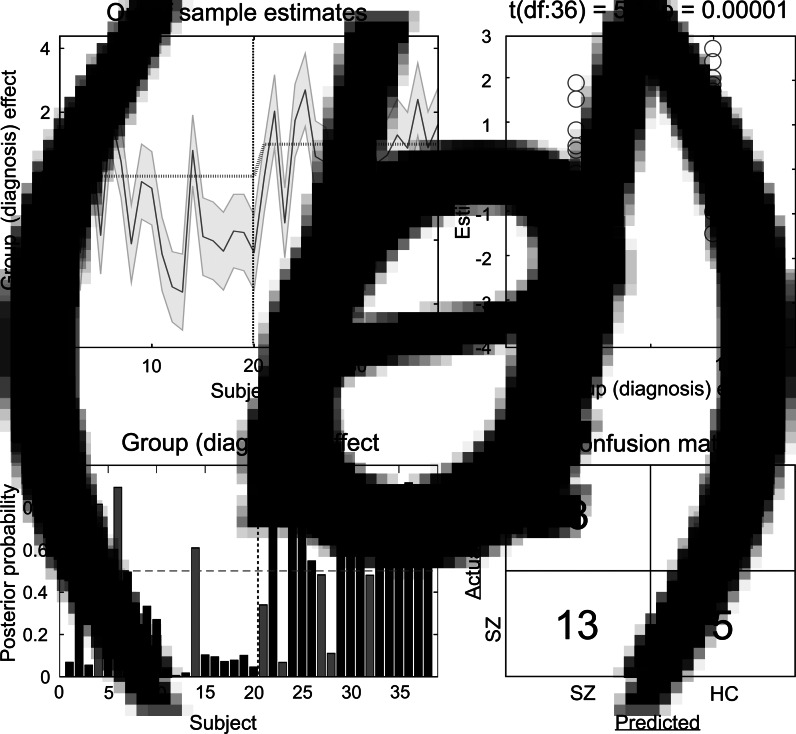
Results of the leave-one-out cross-validation of the DCM. (*a*) Group effect for each subject (dark grey line) with 95% confidence interval (shaded area). First 20 subjects are the controls, while subjects from 21 to 38 are the patients. (*b*) The actual subject effect (0 for controls and 1 for patients) plotted against the expected value of the estimated subject effect (*p* value is from a two-sample *t* test). (*c*) Each subject's posterior probability for belonging to the second group (patients). Vertical dotted line indicates boundary between groups. Black bars indicate posterior predictive densities for correctly classified participants, gray bars indicate incorrect classification. The cut-off value was 0.5 (horizontal dotted line). (*d*) Confusion matrix for the leave-one-out categorization based on the posterior estimates (SZ, patients with schizophrenia; HC, healthy controls).

The results of the LOO cross-validation relying on the three effective connectivity parameters that differed between study groups (rThal to ACC, rThal to lThal, and intrinsic rThal connections) are presented in Fig. 2. Actual group (diagnosis) effect significantly correlated with estimated group effect (Pearson *r* = 0.65, df = 36, *p* = 0.00001). Based on posterior probabilities from the DCM, 85% of controls (i.e. specificity) and 72% of patients (i.e. sensitivity) were classified correctly (overall accuracy = 79%).

### Correlations between effective connectivity and positive/negative psychotic syndrome scores

**Fig. 3.**
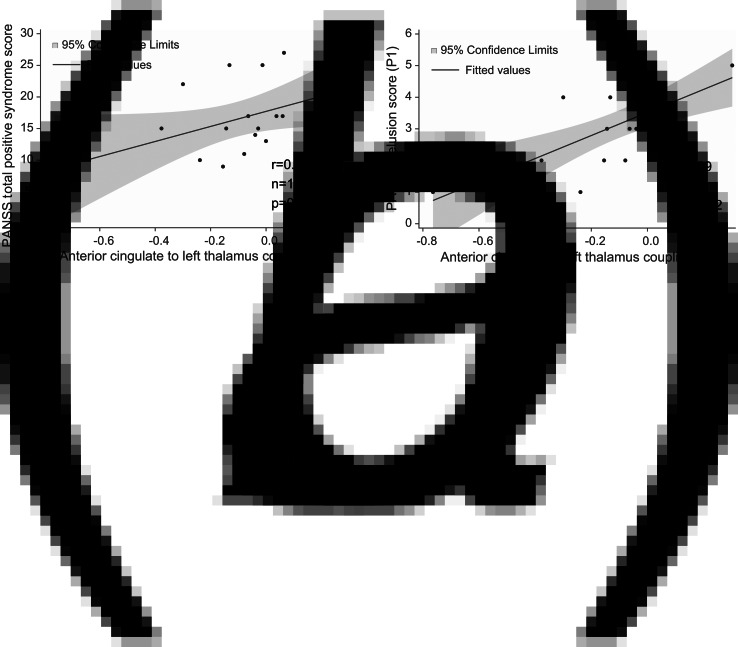
Correlations between effective connectivity measures and positive syndrome scores. (*a*) PANSS total positive syndrome score correlates with ACC→lThal coupling. (*b*) Post-hoc tests showed that among positive PANSS items, delusion score (P1) showed the strongest correlation with ACC→lThal coupling. Linear regression lines and 95% confidence intervals were obtained with linear models; however, statistical results are based on Spearman's non-parametric rank-order correlation tests since there were outliers in the sample.

The strength of effective connectivity from the ACC to the left thalamus correlated significantly with the PANSS total positive syndrome score (Spearman *r* = 0.50, *n* = 17, *p* = 0.04). In order to further explore this result, the correlation between each of the seven items of the positive scale and functional connectivity between ACC and the L-Thal were analyzed post-hoc. Effective coupling correlated significantly with the delusion (Spearman *r* = 0.69, *n* = 17, *p* = 0.002) subscore (Fig. 3), while the correlation with the persecution/suspiciousness (Spearman *r* = 0.53, *n* = 17, *p* = 0.03) subscore did not survive the correction for multiple testing. Other subscores did not correlate with effective coupling. Benzodiazepine dose in terms of clonazepam equivalents did not correlate with effective connectivity (*p* > 0.05).

### DTI analysis and results

RoIs were defined by transforming the 48 regions of the JHU White-Matter Atlas (Hua et al., 2008; Mori et al., 2008; Mori, Wakana, van-Zijl, & Nagae-Poetscher, 2005; Wakana et al., 2007) into each patient's own image-space, using the ‘Get diffusion metrics from ROI labels’ tool of ‘ExploreDTI’. This plugin utilizes the ‘elastix’ (Klein, Staring, Murphy, Viergever, & Pluim, 2010) software for label registration and exports the average DTI-parameter values (MD, FA) for each region. Spatial alignment of the ROI labels was validated by visual inspection. In further analyses, data obtained from 48 ROIs (online Supplementary Table S1) were imported into SAS (SAS 9.4 software, SAS Institute, Cary, NC, USA). A Bonferroni correction for multiple comparisons was applied resulting in a corrected *α*-value of (0.05/48 = ) 0.001.

**Fig. 4.**
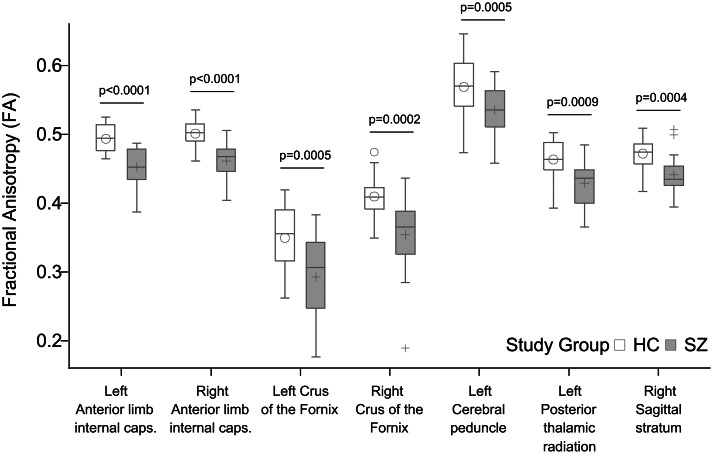
Significant group differences in fractional anisotropy (FA). All *p* values shown survived Bonferroni correction for multiple comparisons.

We found significantly decreased FA in the patient group relative to controls in seven tracts (Fig. 4): anterior limb of the left [Cntrl = 0.49 (s.d. = 0.02), Sch = 0.45 (s.d. = 0.03), *p* < 0.0001] and right [Cntrl = 0.50 (s.d. = 0.02), Sch = 0.46 (s.d. = 0.03), *p* < 0.0001] internal capsule, the left and right stria terminalis and crus of the fornix [Cntrl = 0.35 (s.d. = 0.05), Sch = 0.29 (s.d. = 0.06), *p* = 0.0005 and Cntrl = 0.41 (s.d. = 0.03), Sch = 0.35 (s.d. = 0.06), *p* = 0.0002], the left cerebral peduncle [Cntrl = 0.57 (s.d. = 0.04), Sch = 0.54 (s.d. = 0.04), *p* = 0.0005], the left posterior thalamic radiation [Cntrl = 0.46 (s.d. = 0.03), Sch = 0.43 (s.d. = 0.03), *p* = 0.0009], and the right sagittal stratum [Cntrl = 0.47 (s.d. = 0.02), Sch = 0.44 (s.d. = 0.03), *p* = 0.0004]. PANSS positive and negative scores, antipsychotic dose in terms of chlorpromazine equivalents, and antipsychotic treatment duration did not correlate significantly with FA in any of the RoIs (*p* > 0.05). Mean diffusivity did not differ between study groups after correction for multiple comparisons.

## Discussion

Our current study focused on directed influence within a network comprising the ACC and bilateral MD thalamic nuclei. MD regions have reciprocal and direct connections with the ACC (e.g. Eckert et al., 2012; Klein et al., 2010a), and morphometric neuroimaging studies suggest volume deficit in SZ in MD nuclei (e.g. Byne et al., 2009). We used DCM and DTI to study effective and structural connectivity, respectively, in patients with SZ. We focused on effective connectivity between a prefrontal structure, the ACC, and bilateral thalamus. The ACC has been suggested as a core structure affected by the disorder (Brugger & Howes, 2017) and fronto-thalamic circuitry has long been implicated in SZ (e.g. Murray & Anticevic, 2017; Pergola et al., 2015). It is worth noting that the patterns of prefrontal–thalamic functional connectivity during attentional control have also been suggested as an intermediate phenotype for studying SZ (e.g. Antonucci et al., 2016, 2019). We found decreased effective and structural connectivity in patients compared to neurotypical controls. Effective coupling strength between network nodes correlated with delusion symptom severity. However, we did not find evidence for a relationship between WM connectivity and clinical symptoms.

Previous DCM studies of fronto-thalamic coupling in SZ during a Stroop task found evidence for abnormal effective connectivity. Specifically, Wagner et al. (2013) modeled effective connectivity with DCM and used voxel-based morphometry to study structural deficits. Their results showed lower coupling strength from the ACC to the left and right thalamus and disrupted WM structural connectivity in the fronto-cingulo-thalamic network. In a subsequent study with similar methods, they found evidence for a disrupted fronto-thalamo-cerebellar network (Wagner et al., 2015). Here, we investigated effective connectivity during resting state and found that coupling strength from the right thalamus to the ACC and the right thalamus to the left thalamus was diminished in patients relative to neurotypical controls. Functional network connectivity differs between rest and task, and a meta-analytic study suggests that the thalamus plays a key role in the change of network configurations (Di, Gohel, Kim, & Biswal, 2013). Although network topography remains overall preserved, task demands systematically reconfigure resting-state functional coupling (Gonzalez-Castillo & Bandettini, 2018). Therefore, the most parsimonious explanation for the discrepancy in ACC–thalamic effective connectivity between previous and our current results is that coupling likely differs between task and rest conditions. Thus, effective connectivity during resting state might be altered in SZ in a different way than it is altered during task. While during the task, the influence of ACC over the thalamus is decreased in SZ (Wagner et al., 2013, 2015), our current results suggest that resting state is characterized by a decreased influence of the right thalamus over the ACC in SZ.

In addition to altered ACC–thalamic coupling, we found that the estimates of within-region coupling in the right thalamus were increased in patients relative to controls. Intrinsic connections lend self-inhibitory properties to regions in order to preclude any run-away excitation. We found stronger self-inhibition in the right thalamus in patients relative to controls which indicates a decrease in the gain or excitability of this structure. That is, assuming that the right thalamus receives ACC input of the same strength across the groups, the same input elicits a relatively smaller response from this structure. This is in line with the involvement of the thalamus in SZ and suggests that structural deficit (e.g. Brugger & Howes, 2017; Byne et al., 2009; Dorph-Petersen & Lewis, 2017; Pergola et al., 2015) alters thalamic resting-state activity. Furthermore, our findings are also consistent with the results of Parnaudeau et al. (2013), who found in animal models that a subtle decrease in the medio-dorsal thalamus activity is sufficient to trigger selective impairments in prefrontal-dependent cognitive tasks similar to those in patients with SZ. In addition, we found a decreased inter-thalamic functional connectivity in patients relative to controls. While some previous investigations described the functional connectivity between the left and right thalamus and discussed the significance of increased inter-thalamic functional connectivity in multiple sclerosis (d'Ambrosio et al., 2017; Liu et al., 2015; Stein et al., 2000) less is known about the inter-thalamic connectivity in patients with SZ.

We applied a LOO method to validate functional connectivity-based classification between patients and controls and achieved an overall accuracy of 79%. This result is comparable to the findings of previous studies applying linear discriminants on rsfMRI measures where overall accuracy was in the range of 71% and 84% (Arbabshirani, Kiehl, Pearlson, & Calhoun, 2013; Rashid et al., 2016; Shen, Wang, Liu, & Hu, 2010).

Besides effective connectivity in the ACC–thalamus network, we have studied whole-brain structural connectivity using DTI, a measure of anatomical connectivity through WM tracts. We found altered structural connectivity in patients in regions containing fibers that connect hubs of the ACC–thalamus network. Tracts showing lower FA in patients relative to controls included the anterior limbs of the internal capsule (containing fronto-thalamic and cingulo-thalamic fibers), the crus fornix, the cerebral peduncles, the sagittal striatum, and the posterior thalamic radiation, which findings are in line with the results of previous studies (Fitzsimmons et al., 2013; Zhang et al., 2016) describing connectivity impairments in frontal and temporal regions. A large body of evidence indicates that SZ is characterized by impaired structural connectivity. As far as reduced functional connectivity is considered, it is often accompanied by corresponding reductions of structural connectivity (Fornito & Bullmore, 2015; Nelson, Bassett, Camchong, Bullmore, & Lim, 2017; Schmidt et al., 2014). Our current results, specifically the reduced rThal→ACC effective coupling and decreased FA in the anterior limbs of the internal capsule fit this pattern. Although structural connectivity impairments often show a relationship with symptoms (for a review, see Wheeler & Voineskos, 2014) here we found no correlation between FA measures and PANSS scores.

### Limitations

A major limitation of our current study was the small sample size, which limits the generalization of the results, therefore further investigations applying the same methodology with larger sample sizes are needed to confirm our present findings. While we intended to focus on the medio-dorsal thalamic region during the DCM analysis and the spatial parameters we used did correspond to this region, we note that due to the 7 mm radius of the RoIs, the signal we extracted from both thalami likely contained non-DM activity as well. Furthermore, the ACC ROI we used in the present analysis is located superior relative to the centrum of the ACC according to the functional studies and mainly captures the dorsal area of the ACC including both the Brodmann area 24 and 32 (Marusak et al., 2016). Previous studies (Tomasi & Volkow, 2011) showed that the thalamo-cortical network has a connection with several other hubs, such as the precuneus, motor cortex, and the cerebellum. However, these hubs were excluded from our current analysis due to methodological reasons: including more hubs in the DCM analysis would have increased the number of model parameters dramatically (Friston et al., 2011). It is important to note that there were no separate training and validation sets in the discrimination analysis, which strongly limits the generalization of the results. Therefore, further studies are needed applying separate training and validation datasets to confirm these results. The fact that patients on medication were compared with healthy controls without pharmacotherapy further limits our results, since chronic treatment with antipsychotics may have structural effects on bran tissue and treatment with benzodiazepines can affect functional connectivity. However, we did not find correlations between pharmacotherapy and connectivity. While the two groups did not differ in years of education, in marital status, in living place, or in satisfaction of life, the employment rate was lower among patients, which might indicate a lower socioeconomic status.

## Conclusions

We found that effective and structural connectivity within the ACC–thalamus network is impaired in patients with SZ. Coupling parameter of the ACC→left thalamus connection correlates with the severity of delusion symptoms as measured with PANSS. Whole-brain DTI analysis revealed several WM tracts that showed decreased FA relative to controls with a maximum decrease at regions containing fronto-thalamic and cingulo-thalamic fibers. Overall, our results confirm the involvement of the ACC and the MD thalamic nuclei in SZ. To our knowledge, the present study is the first describing decreased effective connectivity in the ACC–thalamus network in patients with SZ in a resting-state setting.

## Supplementary material

For supplementary material accompanying this paper visit https://doi.org/10.1017/S0033291720000859. click here to view supplementary material
