# Supplementary material for: Fronto-thalamic structural and effective connectivity and delusions in schizophrenia: a combined DTI/DCM study
Source: Psychol Med. 2020 Apr 24;51(12):2083–93. doi: 10.1017/S0033291720000859 (PMC8426148; doi:10.1017/S0033291720000859)
Supplement: Supplementary file 1 [file S0033291720000859sup001.docx]

**Supplement**

**Tensor Fitting and Normalization**

Tensor fitting and the calculation of DTI-scalars:

In order to further reduce the effects of other spatially and temporally varying artifacts inherent to DWI-acquisition (physiologic noise – e.g. cardiac pulsation – or system instabilities) we used the RESTORE (Robust Estimation of Tensors by Outlier Rejection) (Chang *et al.*, 2005) algorithm to fit the diffusion tensor model (Alexander *et al.*, 2007, Basser *et al.*, 1994, Jones and Leemans, 2011).This approach uses iteratively adjusted weights in the nonlinear least-squares fitting of the diffusion tensor in each voxel, achieving convergence faster by identifying and excluding directions with outlier values from the fit. By diagonalization, the eigenvalues ($\lambda_{1},\lambda_{2},\lambda_{3}$) of the tensor, i.e. the principal components of the diffusion profile were calculated. $\lambda_{1}$ is the first eigenvalue that measures diffusivity in the strongest direction, and is commonly referred to as axonal diffusivity, as diffusion tends to be strongest along the white matter fiber pathways. $\lambda_{2}$ and $\lambda_{3}$ are the second and third eigenvalues, measuring diffusivity perpendicular to the main direction.

A voxel-wise DTI-measure, called fractional anisotropy (FA) (Alexander *et al.*, 2011, Basser and Pierpaoli, 1996, Pierpaoli and Basser, 1996) was calculated from the tensor eigenvalues following their well-established definitions:

FA is the normalized standard deviation of the three eigenvalues:

$$FA=\sqrt{\frac{3}{2}\times\frac{{{(\lambda}_{1}-MD)}^{2}+{{(\lambda}_{2}-MD)}^{2}+{{(\lambda}_{3}-MD)}^{2}}{\lambda_{1}^{2}+\lambda_{2}^{2}+\lambda_{3}^{2}}}$$

Spatial normalization of DTI scalar images

In order to examine voxel-level differences in diffusion, the DTI parameter maps had to be ‘normalized’ (i.e. transformed into the same coordinate system) to assure spatial correspondence of brain regions across all subjects. As the diffusion-weighted images were spatially aligned to the T1-weighted ones during the correction steps, the calculated DTI-parameter maps were also in alignment. This allowed the use the high-resolution T1W images to determine the common space, to which all DTI-parameter images needed to be transformed.

We used the DARTEL method (Ashburner, 2007) from the SPM12 toolbox (Friston, 1995) on the T1W images, that is a common approach e.g. in voxel-based morphometry (VBM) studies (Fushimi *et al.*, 2016, Simon *et al.*, 2013). This method creates a ‘template’ image in several iteration steps that is the closest to each individual subject’s anatomy. This way the common coordinate system is study-specific, resulting in more efficient handling of macroscopic anatomical differences (such as possible GM-atrophy), compared to other widely used approaches, for example those utilizing the MNI152 space (Mazziotta *et al.*, 2001).

Once the template image was calculated and the transformations (‘flow fields’) linking each subject’s native space to the common space were determined, we used these transformations on the DTI parameter images (‘warping’).

Originally the ‘warping’ function of DARTEL includes a ‘modulation’ step to account for macroscopic anatomical differences. As the method was developed to examine cortical thickness and structure, when e.g. the transformation means merging three voxels in two, the addition of tissue probability values keeps the information of cortical thickness. However, when working with DTI scalar parameters, this addition (preserving the ‘concentration’) would falsify the original diffusion traits, thereby we omitted the ‘modulation’ option in our processing framework.

The performance of the spatial alignment was assessed by visual inspection and the ‘Check Data Quality’ function of the Computational Anatomy Toolbox (‘CAT12’, an extension to SPM12) (Gaser, 2016). This tool calculates a three dimensional spatial correlation coefficient between images; misaligned data is easily identified by the decreased level of correlation. Three subjects (two controls and one aMCI patient) were removed from the voxel-wise calculations following the corresponding results of the two quality assurance methods. An additional aMCI patient was left out of the calculations who was found to be an outlier, performing significantly worse on each test, thereby biasing the calculations.

References

**Alexander, A. L., Hurley, S. A., Samsonov, A. A., Adluru, N., Hosseinbor, A. P., Mossahebi, P., Tromp do, P. M., Zakszewski, E. & Field, A. S.** (2011). Characterization of cerebral white matter properties using quantitative magnetic resonance imaging stains. *Brain Connect* **1**, 423-46.

**Alexander, A. L., Lee, J. E., Lazar, M. & Field, A. S.** (2007). Diffusion Tensor Imaging of the Brain. *Neurotherapeutics : the journal of the American Society for Experimental NeuroTherapeutics* **4**, 316-329.

**Ashburner, J.** (2007). A fast diffeomorphic image registration algorithm. *Neuroimage* **38**, 95-113.

**Basser, P. J., Mattiello, J. & LeBihan, D.** (1994). MR diffusion tensor spectroscopy and imaging. *Biophys J* **66**, 259-67.

**Basser, P. J. & Pierpaoli, C.** (1996). Microstructural and physiological features of tissues elucidated by quantitative-diffusion-tensor MRI. *J Magn Reson B* **111**, 209-19.

**Chang, L.-C., Jones, D. K. & Pierpaoli, C.** (2005). RESTORE: Robust estimation of tensors by outlier rejection. *Magnetic Resonance in Medicine* **53**, 1088-1095.

**Friston, K. J., Holmes, A. P., Worsley, K. J., Poline, J. B., Frith, C. D., and Frackowiak, R. S. J.** (1995). Statistical parametric maps in functional imaging: A general linear approach. *Hum. Brain Mapp.* **2**, 189–210

**Fushimi, Y., Okada, T., Takagi, Y., Funaki, T., Takahashi, J. C., Miyamoto, S. & Togashi, K.** (2016). Voxel Based Analysis of Surgical Revascularization for Moyamoya Disease: Pre- and Postoperative SPECT Studies. *PLoS One* **11**, e0148925.

**Gaser, C.** (2016). Manual Computational Anatomy Toolbox - CAT12.

**Jones, D. K. & Leemans, A.** (2011). Diffusion tensor imaging. *Methods in Molecular Biology* **711**, 127-44.

**Mazziotta, J., Toga, A., Evans, A., Fox, P., Lancaster, J., Zilles, K., Woods, R., Paus, T., Simpson, G., Pike, B., Holmes, C., Collins, L., Thompson, P., MacDonald, D., Iacoboni, M., Schormann, T., Amunts, K., Palomero-Gallagher, N., Geyer, S., Parsons, L., Narr, K., Kabani, N., Le Goualher, G., Boomsma, D., Cannon, T., Kawashima, R. & Mazoyer, B.** (2001). A probabilistic atlas and reference system for the human brain: International Consortium for Brain Mapping (ICBM). *Philos Trans R Soc Lond B Biol Sci* **356**, 1293-322.

**Pierpaoli, C. & Basser, P. J.** (1996). Toward a quantitative assessment of diffusion anisotropy. *Magn Reson Med* **36**, 893-906.

**Simon, L., Kozak, L. R., Simon, V., Czobor, P., Unoka, Z., Szabo, A. & Csukly, G.** (2013). Regional grey matter structure differences between transsexuals and healthy controls--a voxel based morphometry study. *PLoS One* **8**, e83947.

**Supplementary Table 1**

List of the JHU White-Matter Atlas ROIs used in this study

| ROI name | ROI Size [mm^3^] (mean ± SD) |
| --- | --- |
| Anterior_corona_radiata_L | 6451.06±1244.68 |
| Anterior_corona_radiata_R | 6842.05±1286.50 |
| Anterior_limb_internal_caps_L | 2821.35±477.34 |
| Anterior_limb_internal_caps_R | 2568.10±371.08 |
| Body_of_corpus_callosum | 13407.72±2971.76 |
| Cingulum__cingulate_gyrus__L | 2011.30±277.54 |
| Cingulum__cingulate_gyrus__R | 2344.96±306.14 |
| Cingulum__hippocampus__L | 1064.65±144.79 |
| Cingulum__hippocampus__R | 1008.05±133.97 |
| External_capsule_L | 4463.63±519.64 |
| External_capsule_R | 4174.44±451.22 |
| Fornix_column_and_body_of_fornix | 662.87±202.43 |
| Fornix__cres__Stria_terminalis_L | 984.96±139.84 |
| Fornix__cres__Stria_terminalis_R | 927.57±128.02 |
| Genu_of_corpus_callosum | 7567.71±1540.40 |
| Posterior_corona_radiata_L | 5032.49±1073.88 |
| Posterior_corona_radiata_R | 4950.16±1044.97 |
| Posterior_limb_internal_caps_L | 3092.44±485.74 |
| Posterior_limb_internal_caps_R | 2887.15±373.47 |
| Posterior_thalamic_radiation_L | 5922.31±1357.75 |
| Posterior_thalamic_radiation_R | 5406.78±1279.35 |
| Retrolenticular_part_int_caps_L | 2538.28±560.61 |
| Retrolenticular_part_int_caps_R | 2087.77±378.59 |
| Sagittal_stratum_L | 2274.49±412.72 |
| Sagittal_stratum_R | 2192.66±393.16 |
| Splenium_of_corpus_callosum | 12322.14±2105.66 |
| Superior_corona_radiata_L | 8426.31±1830.68 |
| Superior_corona_radiata_R | 8074.14±1881.53 |
| Superior_fronto_occi_fascicul_L | 544.44±152.18 |
| Superior_fronto_occi_fascicul_R | 504.16±127.28 |
| Superior_longitudinal_fascicul_L | 8723.10±1485.23 |
| Superior_longitudinal_fascicul_R | 7516.35±1322.32 |
| Tapetum_L | 908.75±296.68 |
| Tapetum_R | 859.94±259.40 |
| Uncinate_fasciculus_L | 282.30±45.82 |
| Uncinate_fasciculus_R | 289.03±50.55 |
